# Supplementary material for: Socioeconomic impact of the COVID-19 crisis and early perceptions of COVID-19 vaccines among immigrant and nonimmigrant people living with HIV followed up in public hospitals in Seine-Saint-Denis, France
Source: PLoS One. 2023 Oct 20;18(10):e0276038. doi: 10.1371/journal.pone.0276038 (PMC10588853; doi:10.1371/journal.pone.0276038)
Supplement: S1 Appendix — (DOCX) [file pone.0276038.s001.docx]

# Impact of the Covid crisis on PLWHIV

## General Information

- Date of completion of the questionnaire
- Physician's name
- The information leaflet has been read or given to the patient:
- Gender

If male, specify sexual orientation:

- Date of Birth:
- Year of HIV discovery:
- Did you get the COVID?
- Yes confirmed: specify if ICU or other hospitalization service or no hospitalization
- Probable infection, not confirmed
- No or don't know

### Origin:

| Metropolitan France  |
| --- |
| Europe (excluding France, including Turkey)  |
| North Africa  |
| West Africa (Benin, Burkina Faso, Cape Verde, Ivory Coast, Gambia, Ghana, Guinea, Liberia, Mali, Mauritania, Niger, Nigeria, Senegal, Sierra Leone, Togo)  |
| East Africa  |
| Central Africa (Cameroon, Equatorial Guinea, Gabon, Republic of Congo, Chad, Angola, Central African Republic)  |
| Southern Africa (Botswana Lesotho Namibia South Africa Swaziland)  |
| France overseas  |
| Caribbean outside France  |
| North America  |
| South America  |
| Asia  |
| Other : specify  |

- Date of arrival in France
- Administrative status

| French nationality  | Short-term residence permit : receipt - temporary residence permit - permit for less than one year  |
| --- | --- |
| EU citizen  | No paper  |
| Residence permit 10 years  | Other  |
| Residence permit 1 year or more  |  |

### Current work situation:

| Unemployed since before the crisis  | Partial unemployment  | Permanent contract / Civil servant / Liberal profession without precariousness  | Intermittence, temporary work or precarious self-employment  | Retired  |
| --- | --- | --- | --- | --- |
| Job loss between March 2020 and today  | Maternity or parental leave  | Temporary contract  | Student / In training  | Other  |

## Financial impact

**What is the impact of the crisis on your finances?**

| No  |
| --- |
| Improvement  |
| Decrease in income or loss of income without difficulty in making ends meet  |
| Decrease or loss of income leading to precariousness (difficult end of month, late payment of bills, debt) without food insecurity  |
| Decrease or loss of income leading to food insecurity (leading to not eating for at least one day and/or renewed reliance on food aid)  |
| Other  |

## Administrative Impact

**What is the impact of the crisis at the administrative level?**

| No  |
| --- |
| delay in obtaining or renewing an asylum or residence permit with an impact on activity or income (interruption or non-renewal of a contract)  |
| Delay in obtaining or renewing an asylum or residence permit without impacting on activity or income (extended validity of the receipt or expired residence permit)  |
| Delay in obtaining/renewing an allowance  |
| Delay in processing a family reunification application  |
| Delay in obtaining/renewing social security coverage  |
| Other  |

## Impact on housing

### Housing Type:

| Own housing  |
| --- |
| Hosted (relative, association or center)  |
| In the street  |
| Other  |

**What is the impact of the crisis on housing?**

| No  |
| --- |
| Loss of housing  |
| Overcrowding of housing due to the loss of housing of relatives  |
| Late payment  |
| I have slept at least one night in the street since March 2020  |
| Delay in processing an application for social housing or accommodation attributable to COVID  |
| Other  |

## Impacts on relationships

**You live with :**

| Spouse  |
| --- |
| Children  |
| Spouse and children  |
| Friend, roommate/co-resident  |
| Family  |
| It depends (no stable housing)  |
| Alone  |

**If the answer is alone :**

**Since March 2020:**

| I spent several days without contact with anyone and it weighed on me  |
| --- |
| I spent several days without contact with anyone but it didn't weigh on me  |
| I spent several days without seeing anyone physically but I kept email/tel/WhatsApp contacts or equivalent  |
| I saw one or more adults each day  |

**What is the impact of the crisis on your sexual and/or romantic relationships?**

| Separation during the epidemic  |
| --- |
| Commitment or stabilization of a relationship  |
| Increase in the number of partners  |
| Decrease in the number of partners  |
| No partners since the beginning of the epidemic or before  |
| No partner since the beginning of the epidemic but sex / love life before  |
| No impact  |
| Other  |

## Impact on HIV monitoring

**What is the impact of the health crisis on your HIV monitoring?**

| I have had all my hospital appointments, physically or by teleconsultation, and I have had no difficulty  |
| --- |
| I went to all the scheduled appointments but at least once with a strong fear of getting infected  |
| The hospital has cancelled at least one appointment since March 2020 but it hasn't impacted my treatment  |
| The hospital has cancelled at least one appointment since March 2020 and this has made it difficult for me to get my treatment  |
| I cancelled or missed at least one scheduled hospital appointment because I was too afraid to get infected  |
| I cancelled or missed at least one appointment because I could not return from abroad because of COVID  |
| I cancelled or missed at least one appointment because I was unable to return from abroad unrelated to COVID  |
| Other  |

**If any items in the previous question are answered (except 1)**

**Were you worried that the coronavirus epidemic would jeopardize your HIV follow-up?**

| Yes, and it reminded me of previous experiences, of difficulties in accessing treatment or the care system, and I was afraid of dying of HIV  |
| --- |
| Yes, and it reminded me of previous experiences or fears of not having access to treatment or the care system, but I was not afraid of dying from HIV  |
| Yes, I was afraid of dying of HIV and I had never had a similar fear in the past  |
| Yes, I was afraid of endangering my follow-up for the first time, but I was not afraid of dying  |
| No  |
| Other  |

**How does the crisis affect your treatment?**

| I took my ART as usual (or started it and did not stop)  |
| --- |
| I have missed at least 2 consecutive days of treatment (but < 3 weeks)  |
| I stopped my treatment for more than 3 weeks  |
| I do not take any treatment (Elite controller or before initiation)  |
| Other  |

**Why did you miss days of treatment?**

| I ran out of prescriptions because I hadn't had all my appointments."  |
| --- |
| I had traveled  |
| I forgot  |
| I was discouraged or depressed or just not up to it  |
| I was afraid to go out to get my treatment  |
| Other  |

**Question to the physician: Has the viral load been above the detection threshold at least once during the period March 2020 to the day of the questionnaire, while previously undetectable?**

| Yes with the appearance of resistance mutations  |
| --- |
| Yes with the need to change at least one molecule at least temporarily  |
| Yes without consequences |
| No |

## Impacts on addictions

**How has the crisis affected your drinking?**

| No alcohol or previous withdrawal | Increase in consumption | Stability | Decrease or weaning |
| --- | --- | --- | --- |

If increase, calculate in g/J

**What is the impact of the crisis on your tobacco consumption?**

| No smoking or previous withdrawal | Increase in consumption | Stability | Decrease or weaning |
| --- | --- | --- | --- |

**What is the impact of the crisis on your use of recreational drugs (amphetamines, cocaine)?**

| Never used | Increase in consumption | Stability | Decrease or weaning |
| --- | --- | --- | --- |

**How has the crisis affected your use of sedative drugs (heroin, substitutes)?**

| Never used | Increase in consumption | Stability | Decrease or weaning |
| --- | --- | --- | --- |

## Impacts on weight

### Date of last weight gain before March:

### Last weight before March :

### Weight to date:

**Factors influencing weight (**Question for the physician**):**

| No |
| --- |
| Pregnancy or postpartum during the period |
| Smoking cessation |
| Bariatric surgery |
| Other |

## Psychological impacts

**With this new coronavirus, were you afraid of dying?**

| Yes and the doctor reassured me | Yes, and that fear is still there | No |
| --- | --- | --- |

**Has the coronavirus epidemic brought back fears that you felt when you were diagnosed with HIV?**

| Yes, fear of dying that I had already felt like when I was diagnosed with HIV |
| --- |
| Yes, other fears already experienced during the diagnosis |
| I feel vulnerable because I have a chronic illness, but I don't have any reliving (death anxiety or reliving the anxieties I experienced when I was diagnosed with HIV) |
| It's the isolation of social distancing that reminds me of the isolation I experienced when I was diagnosed with HIV |
| I don't have any particular anxiety, I don't feel particularly at risk from the coronavirus |
| On the contrary, I feel that my experience with HIV has made me stronger/more able to stand back and deal with the current health crisis |
| Other |

**Has the epidemic brought back fears unrelated to your HIV infection?**

| Yes, fears related to situations of war or conflict that I have experienced | Yes, fears related to non-sexual violence that I have experienced |
| --- | --- |
| Yes, fears related to my migratory journey | No |
| Yes, fears related to sexual violence that I have experienced |  |

**Did you feel discouraged?**

| Never | Occasionally | Often |
| --- | --- | --- |

**Have you had dark/suicidal thoughts?**

| Never | Occasionally | Often | Acting on it |
| --- | --- | --- | --- |

**Did you introduce a pill against depression, a sleeping pill or anxiolytic treatment during the period?**

| Yes short period |
| --- |
| Yes, still in progress |
| Usual psychotropic treatment not modified |
| Usual psychotropic treatment reduced or interrupted |
| No |

## Vaccination

**If you were offered the COVID vaccine today:**

| I would accept immediately without hesitation |
| --- |
| I would only accept if my doctor recommended it |
| I could consider vaccination but only if my doctor convinces me verbally, answering all my questions and explaining why he or she thinks I should be vaccinated |
| I will only consider it when we have more experience, I am waiting for more people to be vaccinated |
| I would not accept, even if my doctor recommends it because I am afraid of the side effects because of my HIV |
| I would not accept, even if my doctor recommends it, because I am afraid of the side effects but it has nothing to do with my HIV |
| I would not accept, because I feel at little risk of having COVID or a severe form |
| I would not accept, because I think that I respect the barriers very well and that is enough |
| Other |
