# Supplementary material for: Socioeconomic impact of the COVID-19 crisis and early perceptions of COVID-19 vaccines among immigrant and nonimmigrant people living with HIV followed up in public hospitals in Seine-Saint-Denis, France
Source: PLoS One. 2023 Oct 20;18(10):e0276038. doi: 10.1371/journal.pone.0276038 (PMC10588853; doi:10.1371/journal.pone.0276038)
Supplement: S2 Appendix — (DOC) [file pone.0276038.s002.doc]

# Impact de la crise Covid sur les PvVIH

## Informations Générales

- Date de réalisation du questionnaire
- Nom du médecin
- La notice d’information a été lue ou remise au patient
- Sexe :

Si homme, précisez son orientation sexuelle :

- DDN :
- Année de découverte du VIH :
- Avez-vous eu le COVID ?

Oui confirmé : précisez si SSR/Réa ou autre services d’hospitalisation ou pas d’hospitalisation

Infection probable, non confirmée

Non ou ne sait pas

### Origine :

| France métropolitaine |
| --- |
| Europe (hors France, y compris Turquie) |
| Afrique du nord |
| Afrique de l'ouest (Bénin, Burkina Faso, Cap Vert, Côte d'Ivoire, Gambi, Ghana, Guinée, Libéria, Mali, Mauritanie, Niger, Nigéria, Sénégal, Sierra Léone, Togo) |
| Afrique de l'est |
| Afrique centrale (Cameroun, la Guinée équatoriale, le Gabon, la République du Congo Tchad l’Angola la République centrafricaine) |
| Afrique australe (Botswana Lesotho Namibie Afrique du Sud Swaziland) |
| Outre-Mer |
| Caraïbes hors France |
| Amérique du nord |
| Amérique de sud |
| Asie |
| Autre |

- Date d’arrivée en France
- Situation administrative

| Nationalité française | Titre de séjour court : récépissé - autorisation provisoire de séjour - titre de moins d'un an |
| --- | --- |
| Ressortissant UE | Pas de papier |
| Carte de séjour 10 ans | Autre |
| Titre de séjour 1 an ou plus |  |

### Situation professionnelle actuelle :

| Sans emploi depuis avant la crise | Chômage partiel | CDI / Fonctionnaire / Profession libérale sans précarité | Intermittence, intérim ou auto-entreprenariat précaire | Retraité |
| --- | --- | --- | --- | --- |
| Perte d'emploi entre mars 2020 et aujourd'hui | Congé maternité ou parental | CDD | Etudiant / En formation | Autre |

## Impact financier

**Quel est l'impact de la crise sur vos finances ?**

| Aucun |
| --- |
| Amélioration |
| Diminution de revenus ou perte de revenus sans difficulté à finir les fins de mois |
| Diminution ou perte de revenus entrainant une précarité (fin de mois difficile, retard de paiement de factures, endettement) sans insécurité alimentaire |
| Diminution ou perte de revenus entrainant une insécurité alimentaire (conduisant à ne pas manger au moins une journée et/ou recours nouveau à l’aide alimentaire) |
| Autre |

## Impact Administratif

**Quel est l'impact de la crise au niveau administratif ?**

| Aucun |
| --- |
| retard à l’obtention ou au renouvellement d’un droit d’asile ou d’un titre de séjour avec impact sur l’activité ou les revenus (interruption ou non renouvellement de contrat) |
| retard à l’obtention ou au renouvellement d’un droit d’asile ou d’un titre de séjour sans impact sur l’activité ou les revenus (validité prolongée du récépissé ou du titre de séjour périmé) |
| Retard à l'obtention / renouvellement d'une allocation |
| Retard au traitement d'une demande de regroupement familial |
| Retard à l'obtention / renouvellement d'une protection sociale |
| Autre |

## Impact sur le logement

### Type de logement :

| Logement propre |
| --- |
| Hébergé (proche, association ou centre) |
| A la rue ou SAMU social |
| Autre |

Quel est l'impact de la crise au niveau logement ?

| Aucun |
| --- |
| Perte de logement |
| Surpopulation du logement dû à la perte de logement des proches |
| Retard de paiement |
| J'ai dormi au moins une nuit à la rue depuis mars 2020 |
| Retard au traitement d'une demande de logement social ou d'hébergement attribuable au COVID |
| Autre |

## Impacts sur les relations

Vous vivez avec :

| Conjoint.e |
| --- |
| Enfant.s |
| Conjoint.e et enfant.s |
| Ami.e, colocataire.s/coresident.e.s |
| Famille |
| Ca dépend (pas de logement stable) |
| Seul.e |

**Si la réponse est seul.e**

Depuis mars :

| J'ai passé plusieurs jours de suite sans contact avec personne et ça m'a pesé |
| --- |
| J'ai passé plusieurs jours de suite sans contact avec personne mais ça ne m'a pas pesé |
| J'ai passé plusieurs jours de suite sans voir physiquement personne mais j'ai gardé des contacts mails/tel/WhatsApp ou équivalents |
| J'ai vu un ou plusieurs adultes chaque jour |

**Quel est l'impact de la crise sur vos relations sexuelles et/ou amoureuses ?**

| Séparation pendant l'épidémie |
| --- |
| Engagement ou stabilisation d'une relation |
| Augmentation du nombre de partenaires |
| Diminution du nombre de partenaires |
| Aucun partenaire depuis le début de l'épidémie ni avant |
| Aucun partenaire depuis le début de l'épidémie mais vie sexuelle / amoureuse avant |
| Aucun impact |
| Autre |

## Impact sur le suivi du VIH

**Quel est l'impact de la crise sanitaire sur le suivi de votre VIH ?**

| J'ai eu tous mes rendez-vous hospitaliers, en présentiel ou en téléconsultation et ça ne m'a pas posé de difficulté |
| --- |
| Je suis allé.e à tous les rendez-vous prévus mais au moins une fois avec une forte peur de me contaminer |
| L'hôpital a annulé au moins un rendez-vous depuis mars 2020 mais ça n'a pas eu d'impact sur mon traitement |
| L'hôpital a annulé au moins un rendez-vous depuis mars 2020 et ça m'a mis.e en difficulté pour obtenir mon traitement |
| J'ai annulé ou manqué au moins un rendez-vous prévu à l'hôpital car j'avais trop peur de me contaminer |
| J'ai annulé ou manqué au moins un rendez-vous car je n'ai pas pu rentrer de l'étranger à cause du COVID |
| J'ai annulé ou manqué au moins un rendez-vous car je n'ai pas pu rentrer de l'étranger sans rapport avec le COVID |
| Autre |

**Si réponses à tous les items de la question précédente (sauf la 1)**

**Avez-vous eu peur que l'épidémie de coronavirus ne mette en danger votre suivi VIH ?**

| Oui et ça m'a rappelé des expériences antérieures, de difficultés d'accès au traitement ou au système de soins et j'ai eu peur de mourir du VIH |
| --- |
| Oui et ça m'a rappelé des expériences ou des peurs antérieures de ne pas avoir accès au traitement ou au système de soins, mais je n'ai pas eu peur de mourir du VIH |
| Oui j'ai eu peur de mourir du VIH et je n'avais jamais eu de peur similaire dans le passé |
| Oui j'ai eu peur de mettre en danger mon suivi pour la première fois mais je n'ai pas eu peur d'en mourir |
| Non |
| Autre |

**Quel est l'impact de la crise sur la prise de votre traitement ?**

| J'ai pris mon TARV comme d'habitude (ou je l'ai commencé et je ne l'ai pas interrompu) |
| --- |
| J'ai raté au moins 2 jours consécutifs de traitement (mais < 3 semaines) |
| J'ai arrêté mon traitement plus de 3 semaines |
| Je ne prends pas de traitement (Elite contrôleur ou en amont de l'initiation) |
| Autre |

**Pourquoi avez vous manqué des jours de traitement ?**

| J'ai manqué d'ordonnance parce que je n'avais pas eu tous mes RDV" |
| --- |
| J'avais voyagé |
| J'ai oublié |
| J'étais découragé.e ou déprimé.e ou je n'avais pas la tête à ça |
| J’avais peur de sortir chercher mon traitement |
| Autre |

**Est-ce que la charge virale a été supérieure au seuil de détection au moins une fois sur la période mars 2020 au jour du questionnaire alors qu'indétectabilité antérieure ?**

| Oui avec apparition de mutations de résistance |
| --- |
| Oui avec nécessité de changer au moins une molécule au moins temporairement |
| Oui sans conséquences |
| Non |

Participation du médecin

## Impacts sur les addictions

**Quel est l'impact de la crise sur votre consommation d'alcool ?**

| Pas d'alcool ou sevrage antérieur | Augmentation de la consommation | Stabilité | Diminution ou sevrage |
| --- | --- | --- | --- |

Si augmentation, calculez en g/J

**Quel est l'impact de la crise sur votre consommation de tabac ?**

| Pas de tabac ou sevrage antérieur | Augmentation de la consommation | Stabilité | Diminution ou sevrage |
| --- | --- | --- | --- |

**Quel est l'impact de la crise sur votre consommation de drogues récréatives (dérivés amphétamines, cocaïne) ?**

| Jamais d'usage | Augmentation de la consommation | Stabilité | Diminution ou sevrage |
| --- | --- | --- | --- |

**Quel est l'impact de la crise sur votre consommation de drogues sédatives (héroïnes, produits de substitution) ?**

| Jamais d'usage | Augmentation de la consommation | Stabilité | Diminution ou sevrage |
| --- | --- | --- | --- |

## Impacts sur le poids

### Date de la dernière prise de poids avant mars :

### Dernier poids avant mars :

### Poids à ce jour :

**Facteurs influant le poids :**

| Aucun |
| --- |
| Grossesse ou post-partum sur la période |
| Sevrage tabagique |
| Chirurgie bariatrique |
| Autre |

Question à destination du médecin

## Impacts psychologiques

**Avec ce nouveau coronavirus, avez-vous eu peur de mourir ?**

| Oui et le médecin m'a rassuré.e | Oui et cette peur est toujours là | Non |
| --- | --- | --- |

**L'épidémie de coronavirus vous a-t-elle fait revivre des peurs que vous aviez ressenties comme à l'annonce du VIH ?**

| Oui, peur de mourir que j'avais déjà ressentie comme à l'annonce du VIH |
| --- |
| Oui autres peurs déjà traversées lors du diagnostic |
| Je me sens vulnérable parce que j'ai une maladie chronique, mais je n'ai pas de reviviscence (angoisse de mort ou revival des angoisses traversées à l'annonce du VIH) |
| C'est l'isolement lié aux distanciations sociales qui me rappelle l'isolement dans lequel m'a mis la découverte du VIH |
| Je n'ai pas d'angoisse particulière, je ne me sens pas particulièrement à risque vis à vis du coronavirus |
| J'ai au contraire l'impression que mon expérience du VIH me rend plus fort.e / plus de recul pour affronter la crise sanitaire actuelle |
| Autre |

**L'épidémie vous a-t-elle fait revivre des peurs sans lien avec votre infection par le VIH ?**

| Oui peurs liées à des situations de guerre ou de conflit que j'ai traversées | Oui peurs en lien avec des violences non sexuelles que j'ai vécues |
| --- | --- |
| Oui peurs liées à mon parcours migratoire | Non |
| Oui peurs en lien avec des violences sexuelles que j'ai vécues |  |

**Vous êtes-vous senti découragé.e ?**

| Jamais | Occasionnellement | Souvent |
| --- | --- | --- |

**Avez-vous eu des idées noires / suicidaires ?**

| Jamais | Occasionnellement | Souvent | Passage à l'acte |
| --- | --- | --- | --- |

Avez-vous introduit un traitement antidépresseur, somnifère ou anxiolytique sur la période ?

| Oui courte période |
| --- |
| Oui toujours en cours |
| Traitement psychotrope habituel non modifié |
| Traitement psychotrope habituel diminué ou interrompu |
| Non |

## Vaccination

**Si le vaccin COVID vous était proposé aujourd'hui :**

| J'accepterais tout de suite sans hésiter |
| --- |
| J'accepterais seulement si mon médecin me le recommande |
| Je pourrais envisager la vaccination mais seulement si mon médecin me convainc oralement, en répondant à toutes mes questions et en m'expliquant pourquoi il ou elle pense que je dois être vacciné.e |
| Je l'envisagerai seulement quand on aura plus de recul, j'attends que plus de gens soient vaccinés |
| Je n'accepterais pas, même si mon médecin me le recommande car j'ai peur des effets secondaires à cause de mon VIH |
| Je n'accepterais pas, même si mon médecin me le recommande, car j'ai peur des effets secondaires mais ça n'a rien à voir avec mon VIH |
| Je n'accepterais pas, parce que je me sens peu à risque d'avoir le COVID ou une forme grave |
| Je n'accepterais pas, parce que je pense que je respecte très bien les mesures barrières et que cela suffit |
| Autre |
